# The LC-QTOF-MS/MS analysis data of detected metabolites from the crude extract of *Datura stramonium* leaves

**DOI:** 10.1016/j.dib.2019.104094

**Published:** 2019-06-01

**Authors:** Kudzanai Ian Tapfuma, Lukhanyo Mekuto, Maya Mellisa Makatini, Vuyo Mavumengwana

**Affiliations:** a Department of Biomedical Sciences, Division of Molecular Biology and Human Genetics, Faculty of Medicine and Health Sciences, Stellenbosch University, PO Box 19063, Tygerberg 7505, Cape Town, South Africa; b Department of Chemical Engineering, Faculty of Engineering and the Built Environment, University of Johannesburg, PO Box 17011, Doornfontein, Johannesburg 2028, South Africa; c Molecular Sciences Institute, School of Chemistry, University of the Witwatersrand, P.O Box Wits 2050, Johannesburg, South Africa

**Keywords:** *Datura stramonium*, Metabolite profiling, LC-QTOF-MS/MS

## Abstract

This data article presents the untargeted metabolite profiling of a crude extract from the leaves of *Datura stramonium*. The plant was collected in Johannesburg (South Africa) and the extract was prepared by firstly air-drying fresh *D. stramonium* leaves for one week, grinding the dry leaves into fine powder, followed by solvent extraction using a 1:1 solvent mixture of dichloromethane and methanol (v/v) to extract the compounds. The extract was concentrated at 65 °C to obtain a solid crude extract which was then stored under refrigeration at −80 °C. Qualitative tandem liquid chromatography quadrupole time of flight mass spectrometry (LC-QTOF-MS/MS) was utilized to identify compounds in the extract. The data processing revealed the presence of 76 known compounds in the crude extract from the leaves. This data article contains the m/z [M + H^+^] values, retention times and corresponding database search hit identities of the 76 compounds and the comprehensive list of m/z values detected during the LC-QTOF-MS/MS analysis.

**Table:** Specifications table

Subject area	*Biochemistry*
More specific subject area	*Metabolomics, Natural Products Research, Spectrometry*
Type of data	*Tables and Figures*
How data was acquired	*Data was acquired using liquid chromatography mass spectrometry (LCMS) through a Dionex UltiMate 3000 ultra-high-performance liquid chromatography (UHPLC) (Thermo Scientific, Darmstadt, Germany) coupled to a Compact™ QTOF (Bruker Daltonics, Bremen, Germany)*
Data format	*Raw and Analyzed data*
Experimental factors	*Dried leaves from healthy D. stramonium were extracted with a 1:1 solvent mixture of dichloromethane and methanol (v/v), and concentrated at 65 °C.*
Experimental features	*Untargeted metabolite profiling for D. stramonium leaves was performed*
Data source location	*D. stramonium was collected in Midrand, Johannesburg, South Africa (25°55′48.9″S 28°06′08.9″E); solvent extraction from the leaves was done at the University of Johannesburg, South Africa (26°11′40.2″S 28°03′27.5″E); LC-QTOF-MS/MS analysis and data processing was done at the University of the Witwatersrand (26°11′27″S 28°1′49″E).*
Data accessibility	*Data is within this article. The compounds identified using LC-QTOF-MS/MS (ESI+) analysis are available with this article in*Supplementary Table 1*and the comprehensive list of detected analytes is available in*Supplementary Table 2*.*
Related research article	*J. Y. Won, S.Y. Son, S. Lee, D. Singh, S. Lee, J. Seok Lee* *Strategy for screening of antioxidant compounds from two ulmaceae species based on liquid chromatography-mass spectrometry* *Molecules, 23 (2018), pp. 1–15*[1]

**Table:** 

**Value of the data** • This data provided the untargeted metabolite profiling of compounds that can be expected from the leaves of *D. stramonium*. • This data provides information to researchers of herbal medicinal plants in designing effective drug discovery assays for the discovery of new therapeutic applications of compounds from *D. stramonium*. • This article provides a method for identification of compounds from medicinal plant extracts using untargeted LC-QTOF-MS/MS analysis.

## 1 Data

**Fig. 1:**
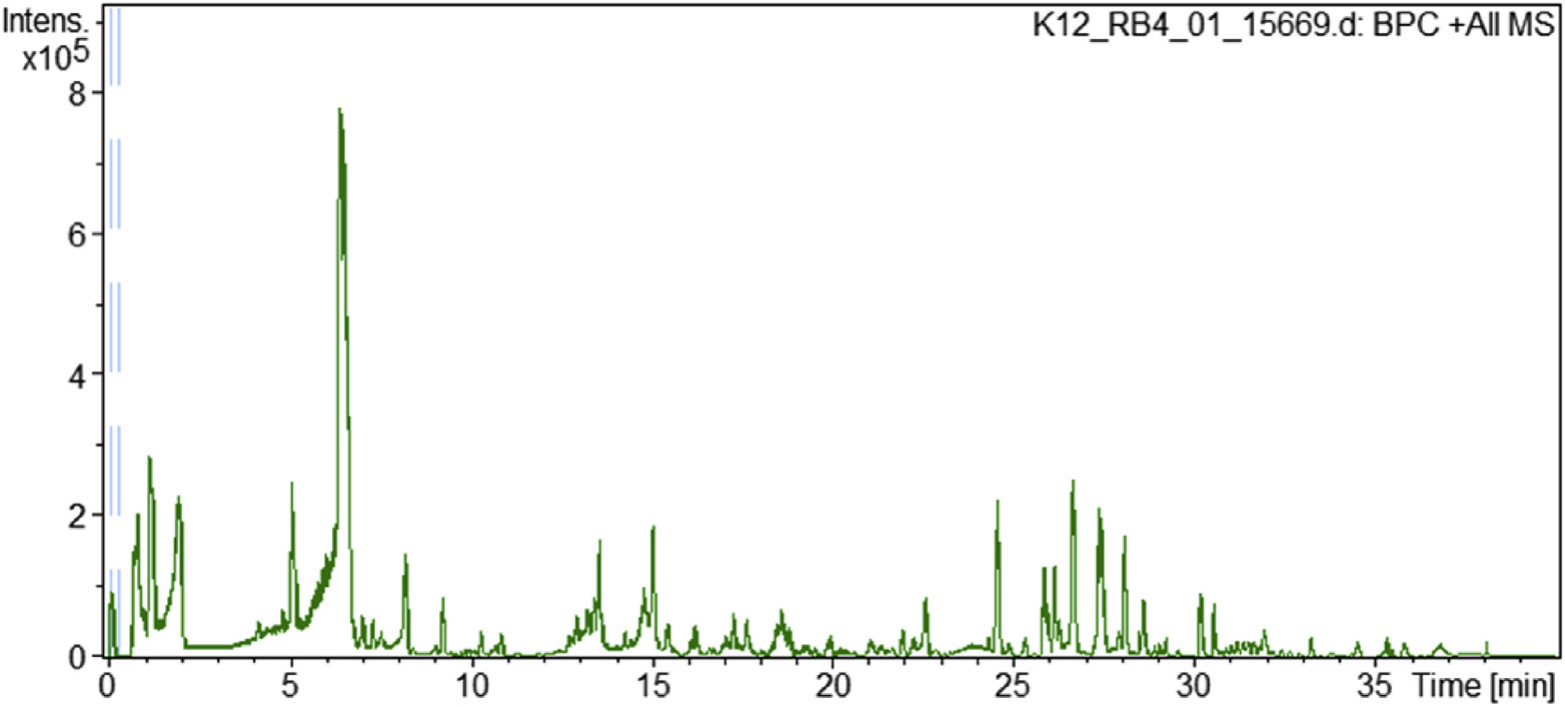
Base peak chromatogram (BPC) of the crude extract from leaves of *D. stramonium.*

Fig. 1 shows the base peak chromatogram of *D. stramonium* which was obtained by analyzing a crude extract from the leaves using LC-QTOF-MS/MS. The data of 76 identified compounds which includes the measured m/z [M + H^+^] values, calculated m/z, calculated mass, calculated mass error (err ppm), retention time (RT min) and the hit identities from the search of three compound databases, namely PubChem, KEGG Compound and ChemSpider is presented in Supplementary Table 1. The raw data of all analytes detected during the LC-QTOF-MS/MS analysis are available in the file Supplementary Table 2.

## 2 Experimental design, materials, and methods

### 2.1 Collection and preparation of plant material

Healthy growing *D. stramonium* was collected from Johannesburg (South Africa) at the following coordinates: 25°55′48.9″S 28°06′08.9″E. The plant sample was immediately transferred to the laboratory after collection and the fresh leaves were allowed to air dry for one week. Extraction of compounds was then done by firstly grinding the leaves using an electric grinder and then mixing 1 L of a 1:1 solvent mixture of dichloromethane and methanol (v/v) with 200 g of ground leaves powder. The powder-solvent mixture was then allowed to shake at 100 rpm for 24 hours upon being filtered through a Whatman No. 2 filter paper and the filtrate concentrated at 65 °C. The resulting solid crude extract was then kept in frozen storage at - 80 °C.

### 2.2 Metabolite profiling of the plant crude extract by LC-QTOF-MS/MS

Metabolite profiling of the crude extract was done using LC-QTOF-MS/MS in positive mode (ESI+). The analysis was done by firstly dissolving 1 mg of the plant extract in 1 mL of HPLC grade methanol followed by sonicating for 10 minutes, and finally filtering through 0.22 μm polyvinylidene fluoride (PVDF) membrane syringe filters into a 1 mL LC auto-sampler vial [2]. A sample injection volume of 5 μL was used for chromatographic separation of analytes in reverse phase ultra-high-performance liquid chromatography (RP-UHPLC) through a Raptor ARC-18 column with dimensions of 2.7 μm (particle size), 2.1 mm (internal diameter), 100 mm (length) and 90 Å (pore size). The analytical run was set at 40 mins and the flow profile of the mobile phase is shown in Table 1. Other parameters of the system are summarized in Table 2.

**Table 1:** Isocratic and gradient flow profiles of the mobile phase.

Time (min)	Flow (μL/min)	Solvent A [0.1% formic acid in H_2_O (v/v)]	Solvent B [0.1% formic acid in acetonitrile (v/v)]	Comment
0–2	300	95%	5%	Isocratic flow
2–30	300	95–5%	5–95%	Gradient flow
30–40	300	5–95%	95–5%	Isocratic flow

**Table 2:** Parameters of the LC-QTOF-MS/MS system.

	Acquisition Parameter
Source type	Electrospray ionization
Ion polarity	Positive
Scan	50–1300 m/z
Set capillary	4500 V
Set end plate offset	−500 V
Set charging voltage	2000 V
Set nebulizer	1.8 Bar
Set dry heater	220 °C
Set dry gas	2.5 L/min
Set APCI heater	0 °C

Data analysis was done using Bruker Compass DataAnalysis software version 4.3 (Bruker Daltonics, Bremen, Germany). MetFrag^1^ web tool version 2.1 was utilized in comparing fragment patterns of fragmented ions with those from compound databases, namely PubChem,^2^ ChemSpider^3^ and KEGG Compound^4^ [3].

## Footnotes

1 https://msbi.ipb-halle.de/MetFragBeta/.

2 https://pubchem.ncbi.nlm.nih.gov/.

3 http://www.chemspider.com/.

4 https://www.genome.jp/kegg/compound/.
